# Cortico-Cerebellar Monitoring of Speech Sequence Production

**DOI:** 10.1162/nol_a_00113

**Published:** 2024-08-15

**Authors:** Snežana Todorović, Jean-Luc Anton, Julien Sein, Bruno Nazarian, Valérie Chanoine, Birgit Rauchbauer, Sonja A. Kotz, Elin Runnqvist

**Affiliations:** Laboratoire Parole et Langage, CNRS–Aix-Marseille Université, Aix-en-Provence, France; Institute of Language, Communication and the Brain, Aix-en-Provence, France; Centre IRM, Marseille, France; INT, CNRS–Aix-Marseille Université, Marseille, France; Department of Neuropsychology and Psychopharmacology, Maastricht University, Maastricht, The Netherlands; Department of Neuropsychology, Max Planck Institute for Human Cognitive and Brain Sciences, Leipzig, Germany

**Keywords:** cerebellum, fMRI, internal modeling, monitoring, speech production

## Abstract

In a functional magnetic resonance imaging study, we examined speech error monitoring in a cortico-cerebellar network for two contrasts: (a) correct trials with high versus low articulatory error probability and (b) overtly committed errors versus correct trials. Engagement of the cognitive cerebellar region Crus I in both contrasts suggests that this region is involved in overarching performance monitoring. The activation of cerebellar motor regions (superior medial cerebellum, lobules VI and VIII) indicates the additional presence of a sensorimotor driven implementation of control. The combined pattern of pre-supplementary motor area (active across contrasts) and anterior cingulate cortex (only active in the contrast involving overt errors) activations suggests sensorimotor driven feedback monitoring in the medial frontal cortex, making use of proprioception and auditory feedback through overt errors. Differential temporal and parietal cortex activation across contrasts indicates involvement beyond sensorimotor driven feedback in line with speech production models that link these regions to auditory target processing and internal modeling-like mechanisms. These results highlight the presence of multiple, possibly hierarchically interdependent, mechanisms that support the optimizing of speech production.

## INTRODUCTION

Speakers monitor their utterances for errors in speech production and rely on a broad cortico-cerebellar network to do so. Specific cognitive mechanisms such as internal modeling, conflict monitoring, and feedback control are all linked to this network (e.g., Gauvin et al., 2016; Hansen et al., 2019a; Okada et al., 2018; Runnqvist, 2023; Runnqvist et al., 2016, 2021; Volfart et al., 2022). Aside from detecting overt acticulation errors (external monitoring), monitoring in speech planning (internal monitoring) can also take place and result in the reduction of contextually inappropriate speech errors; speakers sometimes also swiftly repair errors at a speed that would not be possible if they simply relied on sensory feedback (e.g., Hartsuiker & Kolk, 2001; Levelt, 1983; Motley et al., 1981, 1982; Severens et al., 2012). An inherent assumption of existing speech monitoring models is that both internal and external monitoring rely on the same mechanism(s) applied in different processing stages, before and after articulation (e.g., Levelt et al., 1999; Nozari et al., 2011; Pickering & Garrod, 2013). However, several studies have shown that inner speech and articulated speech differ in error patterns (e.g., Oppenheim & Dell, 2008) and velocity of repair after error detection (Nooteboom & Quené, 2017) and that they display partially different neural correlates of error monitoring (e.g., Hansen et al., 2019a; Okada et al., 2018; Runnqvist et al., 2021). Specifically, previous research has shown dissociations between the regions and mechanisms involved when monitoring is indexed by high error probability contexts in which speakers ultimately produce a correct utterance (i.e., tapping into internal monitoring), as opposed to a context in which overt errors are committed (i.e., also tapping into external monitoring). While both contexts engage the cerebellar Crus I, external monitoring also engages a broader set of medial frontal and temporo-parietal regions and the superior medial cerebellum (SMC; e.g., Gauvin et al., 2016; Hansen et al., 2019a; Loh et al., 2020; Okada et al., 2018; Runnqvist et al., 2021). Consequently, the current study aimed at a better understanding of the differential contributions of different parts of this cortico-cerebellar monitoring network. We wanted to:

1)  assess whether the relevant variable for dissociable brain regions and mechanisms in monitoring is indeed internal versus external monitoring, or rather how distant a linguistic representation is from articulation, or a combination of both;
2)  refine our understanding of the contributions of distinct cerebellar regions to speech production monitoring, ultimately informing theories about functional cerebellar subdivisions; and
3)  gain further insight into the specific functional roles of the medial frontal and parieto-temporal cortices in speech monitoring that are currently ambiguous and subject to much debate.

### Which Variables Are Relevant in Triggering Differentiation of Brain Regions in Monitoring?

Language production is a complex motor skill that involves several processing levels, ranging from semantic retrieval to articulatory programming and execution. Speech errors can originate at any level of processing, resulting in diverse speech errors (e.g., saying “cat” instead of “dog” is a semantic speech error, while saying “gat” instead of “cat” is a phonological or articulatory-phonetic speech error). Some of the processes in speech production only occur during speech planning (semantic retrieval, lexical access) and are thus more cognitive in nature, while others are concommittant to articulation and are thus more motor related. Prior literature assessed internal monitoring at a typically cognitive stage of speech/language processing (pragamtic or lexical; e.g., Hansen et al., 2019b; Runnqvist et al., 2021; Severens et al., 2012; Teghipco et al., 2023). In turn, external monitoring has mostly been assessed by pooling together multiple overt speech errors, including an undeniable yet unknown ratio of motor source (e.g., Gauvin et al., 2016; Runnqvist et al., 2021). Hence, there is a potential confound between internal and external monitoring as opposed to monitoring of cognitive versus motor properties of speech. This confound can be countered in the case of external monitoring by separating overt errors based on their functional sources (e.g., semantic vs. articulatory errors). However, doing so is difficult as overall error rates are often low, even in error-eliciting language production tasks (but see Ganushchak & Schiller, 2008a, 2008b, and McCall et al., 2023).

Here we adopted an alterative approach to address this potential confound by manipulating error probability (high vs. low), allowing to assess different degrees of internal monitoring (high vs. low), of a motor-related variable, namely, syllabification. Concretely, participants produced pseudowords made up of sound combinations that were either novel (phonotactically illegal) or part of their native French language repertoire (phonotactically legal). As in previous studies (e.g., Runnqvist et al., 2021; Severens et al., 2012), the contrast between the more error-prone (illegal) condition and the less error-prone (legal) condition was taken as an index of internal monitoring but at a more motor-related level compared to prior studies. That is, given the illegal condition entails a higher risk of making errors than the legal condition, more monitoring is required to avoid speech errors. Hence, a difference between the two conditions on correct trials necessarily comprises a difference in internal monitoring. Furthermore, as in previous studies (e.g., Gauvin et al., 2016; Riès et al., 2011; Runnqvist et al., 2021), a contrast of correct trials and errors was taken to index external monitoring. As speakers detect their overt errors quite accurately, committing an error involves error detection (e.g., Gauvin et al., 2016; Postma & Noordanus, 1996).

Note that our internal monitoring contrast also likely involved some degree of external monitoring, and similarly the external monitoring contrast involved some degree of internal monitoring. Importantly, the ratio of each monitoring type should differ as in one case speakers manage to avoid errors despite a high error probability (presumably relying more on internal monitoring), while in the other case they do not (presumably relying more on external monitoring). Dissociations between the contrasts of internal and external monitoring would support the hypothesis that they are indeed relevant variables for triggering differential engagement of the monitoring network. Commonalities would instead support the hypothesis that the more relevant dimension is the cognitive to motor continuum.

### What Are the Distinct Cerebellar Contributions to Speech Production Monitoring?

The cerebellum is considered central to the internal modeling of self-produced actions (e.g., Blakemore et al., 2001; Knolle et al., 2013). Parallel to programming actions, we also predict the sensory consequences these actions will result in through an efference copy. In turn, the efference copy inhibits the expected neural response in the relevant part of somatosensory cortex (reafference cancellation), which then leads to an error signal should expected and actual sensory consequences mismatch (e.g., McCloskey, 1981; Wolpert et al., 1995). Importantly, such efference copying can take place internally, before an action becomes overt (e.g., Blakemore & Sirigu, 2003; Desmurget & Grafton, 2000), and is found for motor and mental actions, including language (e.g., Argyropoulos, 2016; Ito, 2008; Lesage et al., 2017; Moberget et al., 2014).

**Figure 1.**
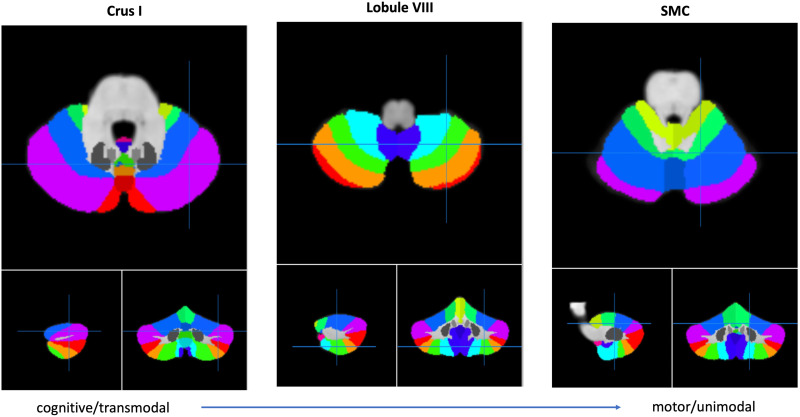
Illustrations of the cerebellum obtained from the SUIT online atlas tool (Diedrichsen et al., 2009). The crosshair in each of the three panels is located at different functional subcompartments (from left to right): the cognitive or transmodal region Crus I, the motor region lobule VIII, and the motor region SMC (superior medial cerebellum).

Some authors have proposed that this internal modeling is being carried out across the whole cerebellum as a unique computation (e.g., Medina & Mauk, 2000; Moberget & Ivry, 2016). This hypothesis was generated indirectly based on the structural and synaptic properties of the cerebellum. In fact, the same type of basic neural circuitry is repeated throughout all cerebellar subdivisions, constituting the fundamental functional module of the cerebellum (e.g., Purves et al., 2001). In turn, this suggests that a single, characteristic computation may be common to all the functions cerebellar circuitry is involved in. A strong candidate for such a unique computation is performance monitoring through internal modeling of self-generated actions (e.g., Ito, 2008; Peterburs & Desmond, 2016). This computation could then be applied to different types of functions/representations in different cerebellar subcompartments. In fact, just as language can be divided into more cognitive and more motor-related parts, the cerebellum has been characterized by cognitive and motor subcompartments, respectively (e.g., Buckner, 2013; Fiez, 1996; Middleton & Strick, 1994; Stoodley & Schmahmann, 2009; Strick et al., 2009). Such functional topography largely corresponds to the so-called double motor representation (lobules VI and VIII) and triple cognitive representation (Crus I, Crus II and lobule IX; e.g., Guell et al., 2018). A different take on linking macrostructure to function are functional gradients (e.g., D’Mello et al., 2020; Guell et al., 2018). By calculating the similarity of intracerebellar resting state functional connectivity patterns, Guell et al. (2018) established a functional gradient, extending bilaterally from lobules IV/V/VI and lobule VIII to posterior aspects of Crus I and II as well as medial regions of lobule IX. The first extreme of the gradient was related to motor function (i.e., modality specific) and the other extreme to functions involving several modalities (i.e., transmodal; see Figure 1). An interesting question explores the relationship between cerebellar anatomy and motor versus non-motor related speech and monitoring functions. Further, a previous study reported that the cerebellar motor region SMC (or lobule IV) was only involved in external monitoring while the cognitive or transmodal region Crus I was involved in both internal and external monitoring.

Two hypotheses can be generated based on these findings. (1) Different compartments, motor and non-motor, might be involved in monitoring motor and non-motor aspects of speech, respectively. If this were the case, manipulating an articulatory motor variable such as phonotactic probability should only activate cerebellar motor compartments, such as SMC and lobule VIII, but not Crus I. (2) Cognitive or transmodal parts of the cerebellum such as Crus I engage in the monitoring of all types of speech related actions, whether motor or cognitive in nature, reflecting that any part of speech production triggers a hierarchically superior type of control. Consequently, Crus I should be implicated regardless of the monitoring type and level of linguistic representation. Here we aimed at contrasting these two hypotheses.

### What Are the Functional Roles of the Medial Frontal and Parieto-Temporal Cortices in Speech Monitoring?

Several studies have observed activation of the medial frontal cortex in external monitoring as indexed by overt speech errors contrasted with correct speech production. Notably, both the pre-supplementary motor area (pre-SMA) and the anterior cingulate cortex (ACC) show differential activation in such circumstances (e.g., Gauvin et al., 2016; Runnqvist et al., 2021). One way to account for this pattern is the concept of conflict-based monitoring (e.g., Gauvin & Hartsuiker, 2020; Nozari et al., 2011). In this framework, conflict itself triggers monitoring in the medial frontal cortex, that, in turn, relays the information to the prefrontal cortex to regulate behavior. This mechanism (e.g., de Zubicaray et al., 1998; Gauvin et al., 2016; Nozari et al., 2011; Riès et al., 2011) would detect conflict at different levels of speech processing (more cognitive and more motor related) and hence in both external and internal error monitoring. However, activation of the pre-SMA in speech production has also been linked to motor-related difficulties, suggesting its role in early stages of motor sequence learning related to the planning and execution of complex speech sequences (Alario et al., 2006). Linking this evidence to that of the pre-SMA in overt error processing, a plausible alternative to conflict monitoring is that this region is involved in monitoring articulatory aspects of production (thus sensorimotor driven). When manipulating an articulatory, motor proximate, variable, this account predicts the engagement of the pre-SMA both for internal monitoring (in situations of high articulatory error probability) and external monitoring (for overt articulatory errors). Regarding the role of the ACC in speech monitoring, an alternative to conflict monitoring could be monitoring of feedback provided via the overt errors (Loh et al., 2020; Runnqvist et al., 2021), where only external monitoring should differentially engage this region (sensorimotor driven).

Finally, there is an ongoing debate about the involvement of temporal and temporo-parietal regions in speech monitoring (e.g., Meekings & Scott, 2021). Traditionally, the detection of speech production errors was considered to exclusively relate to sensory feedback (e.g., Hartsuiker & Kolk, 2001; Levelt et al., 1999; Roelofs, 2020). Either phonologically encoded inner speech or articulated and audible speech would feed into the same speech comprehension loop, leading to the conceptual level of processing, where the appropriateness of an utterance would be monitored. That is, speakers would detect errors by listening to and understanding their own inner and overt speech, similarly to how they listen to and understand someone else’s speech. Because of the central role of speech comprehension in this theory, its hypothesized neural basis is the posterior superior temporal gyrus (pSTG). More recent models of speech production also integrate the temporal (pSTG) and temporoparietal (Sylvian fissure at the temporoparietal boundary, or SPT) in relation to auditory targets and error maps. In these models, mechanisms akin to internal modeling have been implemented as an error signal generated by a mismatch between a (somato-)sensory target and a motor command (e.g., Guenther et al., 2006; Hickok, 2012, 2014; Pickering & Garrod, 2013; Tourville & Guenther, 2011). Furthermore, in these models the cerebellum is hypothesized to contribute to sensory predictions that form the auditory and somatosensory targets, though their role is limited to speech motor function. Hence, these models would also predict pSTG or SPT activation during internal and external monitoring, arguably in combination with cerebellar activation. Indeed, there is converging evidence that the pSTG engages in external monitoring as indexed by articulated, though not necessarily audible speech, overt speech errors, and halted speech (e.g., Hansen et al., 2019a; Okada et al., 2018; Runnqvist et al., 2021). Note that some studies manipulating auditory feedback (e.g., Hirano et al., 1997) or looking into auditory hallucinations (e.g., Shergill et al., 2000) also link pSTG to internal monitoring (e.g., Indefrey & Levelt, 2004). Nonetheless, these studies varied in their use of auditory variables. It is unclear though whether the pSTG activation is actually reflecting internal monitoring, especially as studies explicitly controlling for auditory variables do not seem to observe pSTG in conditions taxing internal monitoring (e.g., Hansen et al., 2019a; Runnqvist et al., 2021). Here we wanted to shed further light on the functional role of temporal and parietal regions by assessing their potential involvement in internal and external monitoring.

### Cerebellar and Cortical ROI Definition in the Current Study

To zoom in on the monitoring network of interest, we used a region of interest (ROI) approach, in which seven ROIs within the cerebellum, medial frontal, temporal, and temporo-parietal cortices were examined in two contrasts. Six of these regions were taken from Runnqvist et al. (2021) and one from Rauschecker et al. (2008; see Table 1 in the next section). As speech is assumed to be left-lateralized in cortex and right-lateralized in the cerebellum, we limited the cortical ROIs to the left and the cerebellar ROIs to the right hemsiphere. In the cerebellum, we chose three right hemispheric regions: Crus I on the transmodal side of Guell et al. (2018) functional gradient, the phylogenetically older SMC, and the phylogenetically younger posterior lobule VIII on the motor side of the gradient. In addition, we chose two regions in the medial frontal cortex (left ACC and pre-SMA) and two regions in temporal and temporo-parietal cortex (left pSTG and SPT). This selection sheds light on potential dissociations across internal and external monitoring for the articulatory variable of interest. Complementing the ROI analyses, we also examined the differential activations related to internal and external monitoring contrasts in broader anatomical ROIs (cerebellum, medial frontal cortex, and temporo-parietal cortex) in a whole brain analysis.

**Table 1.** MNI coordinates and references of the ROI classified by anatomical regions.

Broad anatomical region	Anatomical subregion	MNI coordinates
Cerebellum	R Crus I	(38, −64, −30) Stoodley and Schmahmann, 2009*
	R SMC	(16, −59, −23) Golfinopoulos et al., 2010*
	R posterior lobule VIII	(28, −56, −54) Rauschecker et al., 2008
Medial frontal cortex	L ACC	(−6, 20, 34) Gauvin et al., 2016
	L Pre-SMA	(−6, 8, 49) Gauvin et al., 2016
Temporo-parietal cortex	L pSTG	(−65, −33, 14) Golfinopoulos et al., 2010*
	L SPT	(−54, −30, 14) Okada and Hickok, 2006

*Note*. R = right, L = left. Asterisks indicate meta-analysis or model-based coordinates. SMC = superior medial cerebellum; ACC = anterior cingulate cortex; pre-SMA = pre-supplementary motor area; pSTG = posterior superior temporal gyrus; SPT = sylvian fissure at the parieto-temporal boundary; MNI = Montreal Neurological Institute.

## MATERIALS AND METHODS

### Participants

The study received ethical approval (filed under Id 2017-A03614-49 from the regional ethical committee, Comité de protection des personnes sud Méditerranée I). Twenty-six (17 females) right-handed native speakers of French participated in exchange for monetary compensation. Two participants (1 female, 1 male) were excluded from further analyses due to excessive head movements during data acquisition. The average age of the remaining 24 participants was 26.2 (*SD* 3.5). No participant reported any history of language, neurological, or hearing disorders.

### Materials

Target stimuli were 36 CCVCC syllables (C = consonant, V = vowel) composed of French phonemes. In one half of the stimuli, these phonemes were combined in a phonotactically legal way in French, and their production therefore required less monitoring, even if the syllable was meaningless. The other half of the stimuli were syllables consisting of novel combinations of phonemes (phonotactically illegal), requiring increased monitoring as their production had to be learned (e.g., Segawa et al., 2015). Two-consonant clusters were selected from the French language database lexique.org (New et al., 2001) and filtered for their frequency of occurrence at the beginning or at the end of the syllable (New & Spinelli, 2013). The clusters with high frequency (141.6 ± 251.6) were used to form low monitoring load stimuli, and those with a frequency close to zero (0.3 ± 0.8) to form the high monitoring load ones. The resulting syllables were checked for orthographic neighbors using WordGen software (Duyck et al., 2004). Only one stimulus had one orthographic neighbor (spald), the rest of stimuli had none.

All stimuli were recorded by a Serbian speaker, as Serbian allows pronunciation of all used combinations. Stimuli were also visually presented according to French orthographic rules. As this experiment was part of a larger study that took place over two sessions, any given participant was only presented with half of the stimuli in a counterbalanced manner. Each stimulus was presented 25 times over five experimental runs. The order of the stimuli was pseudorandomized in eight different lists so that the same category (high or low monitoring load) was not presented more than twice in a row and that each stimulus was presented *N* times before any other stimulus was presented *N* + 1 times. Thus, each participant was presented with 225 (25 × 9) high monitoring load and 225 low monitoring load syllables.

### Procedure

**Figure 2.**
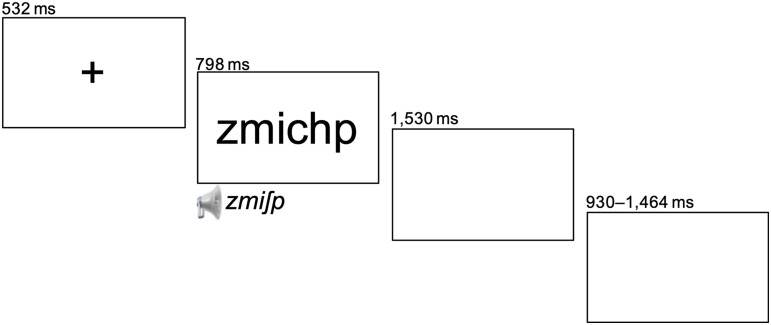
Depiction of an experimental trial.

Syllables remained on the screen for 798 ms and were synchronously presented auditorily for 660 ms. They were preceded by a fixation cross for 532 ms and followed by a blank screen during which the participants were instructed to pronounce the presented syllable. The time for pronunciation was 1,530 ms. The jittered interstimulus-interval was 930 to 1,464 ms. In sum, on average a single trial lasted 4.059 seconds, and the experimental runs lasted 6 minutes each. Figure 2 shows an exemplary trial. Stimulus presentation was controlled by a custom-made software compiled using the LabVIEW development environment (National Instruments) and OptoActive audio system (OptoAcoustics). This software also allowed the recording of vocal productions: Trials were recorded and labeled independently using a multifunction NI PCIe-6353 DAQ (National Instruments) and the FOMRI-III optical noise canceling microphone (OptoAcoustics). Each trial was saved in an independant 16-bit WAV audio file.

### MRI Data Acquisition

Data were collected on a 3-Tesla Siemens Prisma scanner at the Marseille MRI Center using a 64-channel head coil. Functional images covering the whole brain, including the cerebellum, were acquired during task performance, using a multiband blood oxygen level dependent (BOLD)-sensitive gradient echo planar imaging (EPI) sequence (Moeller et al., 2010) (repetition time [TR] = 1,224 ms, echo time [TE] = 30 ms, flip angle = 66°, 54 slices with a thickness of 2.5 mm, field of view [FOV] = 210 × 210 mm^2^, matrix = 84 × 84, slice thickness = 2.5 mm, multiband factor = 3). Whole-brain anatomical magnetic resonance imaging (MRI) data were acquired using high-resolution structural T1-weighted image (MPRAGE sequence, voxel size = 1 × 1 × 1 mm^3^, data matrix 256 × 256, TR/TI (inversion time)/TE = 2,300/900/2.98 ms, flip angle = 9°). Prior to functional imaging and to correct functional image for susceptibility induced distortions, a pair of spin-echo EPI sequences with the same spatial parameters as the BOLD images was acquired twice with opposite phase encode directions along the anterior-posterior axis with the following parameters: TR/TE = 7,060/59 ms, voxel size = 2.5 × 2.5 × 2.5 mm^3^, slices = 54, FOV = 210 × 210 mm^2^. Participants’ movements during data acquisition were controlled using Framewise Integrated Real Time MRI Monitoring (Dosenbach et al., 2017).

### Behavioral Data Processing and Analyses

All syllable productions were transcribed and labeled as incorrect if they contained insertions, omissions, hesitations, or self-repairs, if they were impartial or missing, or if a syllable was pronounced as two or more syllables. They were labeled as correct in all other cases.

### Image Processing and Analyses

Results derive from preprocessing, using fMRIPrep Version 20.2.0 (Esteban et al., 2019; Esteban, Markiewicz, Goncalves, et al., 2020), which is based on Nipype Version 1.5.1 (Esteban, Markiewicz, Burns, et al., 2020; Gorgolewski et al., 2011). A singularity image for fMRIPrep was used and it was run on a cluster of the Mesocentre platform.

#### Anatomical data preprocessing

The T1-weighted (T1w) image was corrected for intensity non-uniformity (INU) with N4BiasFieldCorrection (Tustison et al., 2010), distributed with ANTs Version 2.3.3 (Avants et al., 2008), and used as T1w-reference throughout the workflow. The T1w-reference was then skull-stripped with a Nipype implementation of the antsBrainExtraction.sh workflow (from ANTs), using OASIS30ANTs as target template. Brain tissue segmentation of cerebrospinal fluid (CSF), white matter (WM) and gray matter (GM) was performed on the brain-extracted T1w using FAST (FSL 5.0.9; Zhang et al., 2001). Volume-based spatial normalization to one standard space was performed through nonlinear registration with antsRegistration (ANTs 2.3.3), using brain-extracted versions of both T1w reference and the T1w template. The following template was selected for spatial normalization: ICBM 152 Nonlinear Asymmetrical template version 2009c (Fonov et al., 2009), TemplateFlow ID: MNI152Nlin2009cAsym (Esteban et al., 2022).

#### Functional data preprocessing

For each of the five BOLD runs found per participant (across all sessions), the following preprocessing was performed. First, a reference volume and its skull-stripped version were generated by aligning and averaging one single-band reference (SBRefs). A B0-non-uniformity map (or *fieldmap*) was estimated based on two (or more) EPI references with opposing phase-encoding directions, with 3dQwarp (Cox & Hyde, 1997; AFNI 20160207). Based on the estimated susceptibility distortion, a corrected EPI reference was calculated for a more accurate co-registration with the anatomical reference. The BOLD reference was then co-registered to the T1w reference using FLIRT (FSL Version 5.0.9; Jenkinson & Smith, 2001) with the boundary-based registration (Greve & Fischl, 2009) cost-function. Co-registration was configured with 9 *df* to account for distortions remaining in the BOLD reference. Head-motion parameters with respect to the BOLD reference (transformation matrices and six corresponding rotation and translation parameters) were estimated before any spatiotemporal filtering using MCFLIRT (FSL Version 5.0.9; Jenkinson et al., 2002). The BOLD time series were resampled onto their original, native space by applying a single, composite transform to correct for head-motion and susceptibility distortions. These resampled BOLD time series are referred to as *preprocessed BOLD in original space*, or just *preprocessed BOLD*. The BOLD time series were resampled into standard space, generating a *preprocessed BOLD run in MNI152Nlin2009cAsym space*. First, a reference volume and its skull-stripped version were generated using a custom methodology of fMRIPrep. Several confounding time series were calculated based on the preprocessed BOLD: framewise displacement (FD), DVARS (i.e., spatial standard deviation of successive difference images, e.g., Smyser et al., 2011), and three region-wise global signals. FD was computed using two formulations following Power et al. (2014; absolute sum of relative motions) and Jenkinson et al. (2002; relative root mean square displacement between affines). FD and DVARS are calculated for each functional run, both using their implementations in Nipype (following the definitions by Power et al., 2014). The three global signals are extracted within the CSF, the WM, and the whole-brain masks. Additionally, a set of physiological regressors were extracted to allow for component-based noise correction (CompCor; Behzadi et al., 2007). Principal components were estimated after high-pass filtering the preprocessed BOLD time series (using a discrete cosine filter with 128 s cut-off) for anatomical CompCor (aCompCor). For aCompCor, two probabilistic masks (CSF, WM) were generated in anatomical space. The implementation differs from that of Behzadi et al. (2007). Instead of eroding the masks by 2 pixels on BOLD space, the aCompCor masks were subtracted from a mask of pixels that likely contain a volume fraction of GM. This mask was obtained by thresholding the corresponding partial volume map at 0.05, and it ensured components were not extracted from voxels containing a minimal fraction of GM. Finally, these masks were resampled into BOLD space and binarized by thresholding at 0.99 (as in the original implementation). For each CompCor decomposition, the 12 components with the largest singular values were retained. The head-motion estimates calculated in the correction step were also placed within the corresponding confounds file. The confounded time series derived from head-motion estimates and global signals were expanded with the inclusion of temporal derivatives and quadratic terms for each (Satterthwaite et al., 2013). All resamplings were performed with *a single interpolation step* by composing all the pertinent transformations (i.e., head-motion transform matrices, susceptibility distortion correction, and co-registrations to anatomical and output spaces). Gridded (volumetric) resamplings were performed using antsApplyTransforms, configured with Lanczos’s interpolation to minimize the smoothing effects of other kernels (Lanczos, 1964).

The preprocessed data were imported to and analyzed using the Statistical Parametric Mapping software (SPM12; https://www.Fil.ion.ucl.ac.uk/spm/software/spm12/) in MATLAB R2018b (Mathworks Inc., Natick, MA). Smoothing was performed with an isotropic Gaussian kernel (full-width at half-maximum = 5 mm). Regressors of no interest included 24 head movement regressors, the mean signal of the CSF and WM mask, and 24 aCompCor regressors related to CSF and WM.

For the univariate whole brain analysis, we created a general linear model (GLM) for each participant. The GLM included, for each of the five runs, the following regressors of interest: AnyLoad_Incorrect (corresponding to all overtly committed errors), HighLoad_Correct (corresponding to the correctly produced phonotactically illegal pseudowords), LowLoad_Correct (corresponding to the correctly produced phonotactically legal pseudowords). Regressors of interest were convolved with the canonical hemodynamic response function. Functional data were filtered with a 128 s high-pass filter. For each participant, we estimated the two contrasts: AnyLoad_Incorrect (corresponding to all overtly committed errors) versus HighLoad_Correct and LowLoadCorrect (corresponding to all correctly produced pseudo words) [2 −1 −1] and HighLoad_Correct (corresponding to the correctly produced phonotactically illegal pseudowords) versus LowLoad_Correct (corresponding to the correctly produced phonotactically legal pseudowords) [0 1 −1]. These two contrasts were then explored at the group level with a random effect analysis. All statistical comparisons were performed with a voxelwise threshold of *p* < 0.001 and a cluster extent threshold of 25 voxels.

For the univariate analysis of ROIs, seven anatomical ROIs were created based on the previous literature (Table 1). Spheres with a Montreal Neurological Institute (MNI) coordinates center and a 10 mm radius were created using the MarsBar SPM toolbox (Brett et al., 2002) and for each participant a mask was created by keeping only voxels within each sphere whose GM probability content (as determined by fMRIPrep segmentation) was higher than 0.2. For a given ROI mask and on the basis of unsmoothed functional images, we extracted the mean of the beta value differences between the contrasted experimental conditions as specified above. For each ROI, we performed one-tailed one-sample *t* tests comparing the distribution of the beta value differences to the null hypothesis (no difference). Bonferroni correction was used to correct for multiple comparisons.

## RESULTS

**Figure 3.**
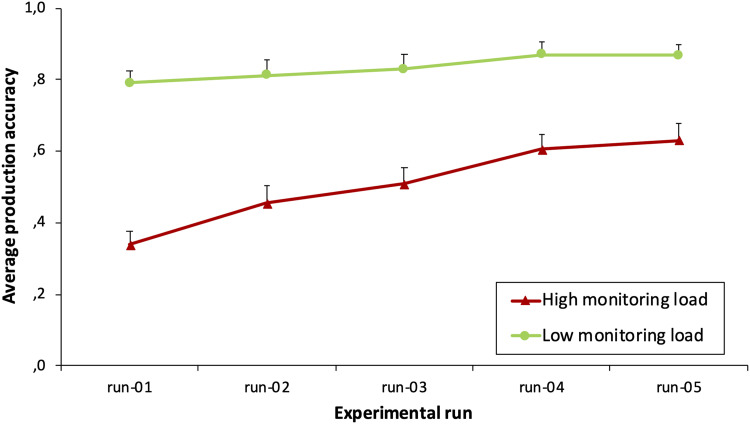
Average production accuracy over the five experimental runs in each monitoring load condition. Error bars represent standard errors of the mean.

Out of the 10,800 syllable productions of all participants, 3,548 were marked incorrect (32.8%, mean standard error [*MSE*] 0.0, *SD* 0.5), of which 2,655 (49.2%, i.e., 2,655/5,400, *MSE* 0.0, *SD* 0.5) were found in the high monitoring load condition and 893 (16.5%, i.e., 893/5,400, *MSE* 0.0, *SD* 0.4) in the low monitoring load condition. This validates that the syllables containing new consonant clusters were more error prone and required more monitoring. A generalized linear mixed model further showed that an interaction between the monitoring load and the number of times an item had been pronounced plays a significant role in predicting production accuracy (*p* < 0.001) and indicates more learning in the high monitoring load condition. Finally, the two conditions differed even in the last experimental run (*p* = 2.84e^−40^), indicating that the monitoring load remained high for the new consonant combinations throughout the experiment. The results are summarized in Figure 3.

**Figure 4.**
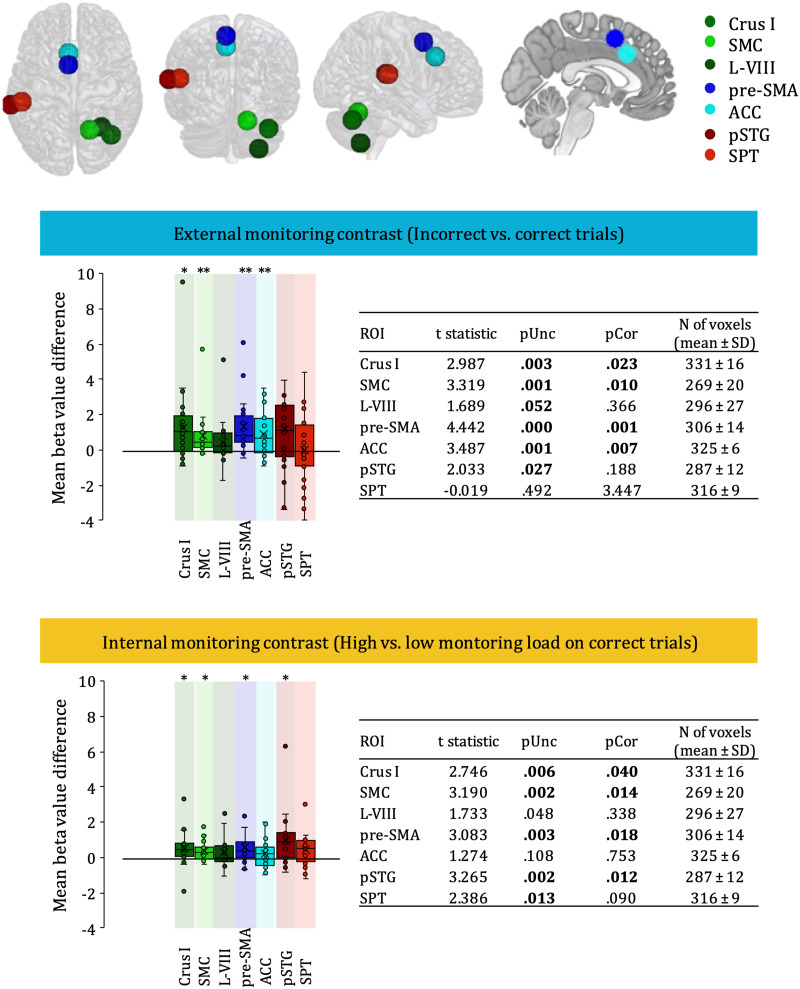
Mean beta value differences in the predefined region of interest (ROI). Top panel represents the locations of the seven predefined regions of interest (ROIs): Crus I, SMC, L-VIII, pre-SMA, ACC, pSTG, and SPT. Middle panel and bottom panel represent the internal monitoring contrast and the external monitoring contrast, respectively. ROIs in medial frontal cortex are represented with blue tones, ROIs in the cerebellum with green tones, and ROIs in temporo-parietal cortex with red tones. The asterisks indicate significant effects <0.05 (*) or <0.005 (**) after applying Bonferroni correction for multiple comparisons. SMC = superior medial cerebellum, L-VIII = lobule VIII, pre-SMA = pre-supplementary motor area, ACC = anterior cingulate cortex, pSTG = posterior superior temporal gyrus, and SPT = Sylvian fissure at the temporoparietal boundary.

Using an ROI approach, we examined the beta value differences in seven predefined regions located in the cerebellum, medial frontal, temporal, and temporo-parietal cortices (Table 1) in two contrasts of interest, namely, external monitoring, contrasting incorrect trials with the correct ones, and internal monitoring, contrasting high and low monitoring load on correctly produced trials (Figure 4). Two cerebellar regions, right Crus I and right SMC, were involved both in the contrast targeting external and internal monitoring of speech sequence production (Crus I, internal monitoring: *p* = 0.04, external monitoring: *p* = 0.02; SMC, *p* = 0.01 for each contrast). This was also the case for the pre-SMA in the left medial frontal cortex (*p* = 0.02 in internal and 0.001 in external monitoring). Internal monitoring was further linked to the left pSTG (*p* = 0.01), while external monitoring was linked to the left ACC (*p* = 0.007).

**Figure 5.**
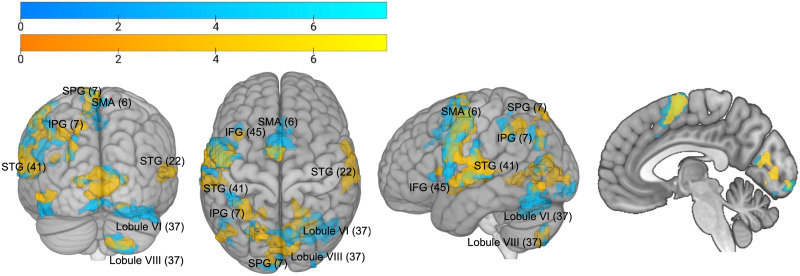
Results of the whole brain analysis. BOLD response of internal monitoring (high vs. low monitoring load for correct trials, yellow) and external monitoring (errors vs. correct trials, blue) contrasts. Overlap across contrasts is visible as green. Statistical *t* maps are overlaid on an MNI template using a voxelwise threshold of *p* < 0.001 and an extent threshold of 25 voxels. Numbers in brackets represent Brodmann areas. IFG = inferior frontal gyrus, IPG = inferior parietal gyrus, SMA = supplementary motor area, SPG = superior parietal gyrus, STG = superior temporal gyrus.

To examine the specificity of the findings from the ROI analyses, we also conducted a whole-brain analysis (Table 2 and Figure 5). After correcting for multiple comparisons, both monitoring contrasts revealed activation in cerebellar lobules VI and VIII, as well as SMA in medial frontal cortex and BA22 in temporal cortex. The internal monitoring contrast further confirmed BA 41 activation in the temporal cortex. Both the internal and the external monitoring contrasts also revealed significant clusters in the parietal cortex as well as in the insula. Table 3 summarizes all the analyses that were carried out.

**Table 2.** Results of the whole-brain analyses of the BOLD response of the (A) external and (B) internal monitoring contrasts.

Region label	Cluster extent	*t* value of the local maximum	MNI coordinates		
			*x*	*y*	*z*
A. External monitoring (errors vs. correct trials)					
L precentral gyrus	760	6.27	−56	8	36
L postcentral gyrus	73	5.64	−69	−15	24
L supplementary motor area	430	6.18	−2	5	59
L middle temporal gyrus	99	5.55	−62	−10	−1
L insula/inferior frontal gyrus, triangular part	32	4.99	−36	25	2
L inferior parietal gyrus	124	5.62	−36	−45	39
L superior occipital gyrus	96	4.92	−22	−68	39
L inferior occipital gyrus	170	6.05	−46	−68	−16
R inferior occipital gyrus	27	4.58	28	−98	−11
L calcarine fissure	92	6.24	−6	−98	−8
R calcarine fissure	240	5.59	21	−68	6
R cerebellum (VI)	414	6.46	26	−65	−24
R cerebellum (VI)	27	5.51	8	−72	−14
R cerebellum (VIII)	145	5.40	24	−72	−54

B. Internal monitoring (phonotactically illegal vs. legal items on correct trials)					
L inferior frontal gyrus, triangular part	34	5.27	−36	18	9
L supplementary motor area	200	6.13	−6	0	64
L superior temporal gyrus	1120	7.36	−64	−18	9
R temporal pole (superior temporal gyrus)	247	7.91	61	2	−8
L superior parietal gyrus	51	5.12	−16	−78	59
L inferior parietal gyrus	163	5.79	−32	−45	44
L middle occipital gyrus	63	5.26	−26	−72	32
L inferior occipital gyrus	104	5.69	−44	−68	−6
L calcarine fissure	662	6.23	−16	−68	9
L lingual gyrus	39	4.38	−14	−55	−1
R cerebellum (VI)	90	5.50	28	−65	−21
R cerebellum (VIII)	154	5.67	21	−72	−54

*Note*. Local maxima of blood oxygen level dependent (BOLD) response separated by >8 mm. Regions were automatically labeled using the automated anatomical labeling atlas 3 (Rolls et al., 2020) in SPM. *X*, *y*, *z* = MNI coordinates in the left-right, anterior-posterior, and inferior-superior dimensions, respectively. All peaks are significant at a voxelwise threshold of *p* < 0.001 (extent threshold = 25 voxels) and at a cluster threshold of *p* < 0.05 with a false discovery rate correction for multiple comparisons. L = left, R = right, MNI = Montreal Neurological Institute.

**Table 3.** Summary of the different analyses conducted.

	Analysis	Contrasted variables	Purpose
Behavioral data	Generalized linear mixed model on production accuracy	High vs. low load monitoring items over five runs	Validate monitoring load assumption underlying imaging contrast
fMRI data	Univariate ROI analysis on BOLD response	Errors vs. correct trials	Index external monitoring
		Correct high vs. correct low monitoring load trials	Index internal monitoring
	Univariate whole brain analysis on BOLD response	Errors vs. correct trials	Assess specificity of ROI findings for external and internal monitoring contrasts
		Correct high vs. correct low monitoring load trials	

*Note*. fMRI = functional magnetic resonance imaging; ROI = region of interest; BOLD = blood oxygen level dependent.

## DISCUSSION

The aim of the current study was to investigate the cortico-cerebellar correlates of error monitoring in the speech production of novel syllable sequences. A postlexical variable, phonotactic legality, was manipulated in two conditions that differed in their degree of required monitoring. The critical condition consisted of novel syllable sequences that had to be learned, hence were heavily monitored. The baseline condition consisted of known syllable sequences, hence required less monitoring. Contrasting these two conditions for correct trials provided an index of internal monitoring at the postlexical level. Contrasting errors with correct production indexed external monitoring. In the following, we first summarize the results that were obtained for each broad anatomical ROI and then discuss the implications of these results regarding the relevant variables driving the recruitment of different parts of the cortico-cerebellar monitoring network.

### Transmodal and Sensory-Motor Driven Monitoring in Distinct Cerebellar Regions Are Both Triggered in Novel Sound Sequence Production

An important goal of the present study was to better understand how distinct cerebellar regions contribute to speech error monitoring. We were interested in highlighting different cerebellar subcompartments as a proxy for internal modeling. Specifically, we contrasted the hypothesis that cerebellar motor regions are engaged in monitoring of motor behavior (including speech motor aspects) and cerebellar cognitive regions are engaged only in cognition (including cognitive aspects of speech), with the idea that any aspect of speech production would trigger activation of the cognitive or transmodal parts of the cerebellum. In line with the latter, an ROI known to be implicated in a broad number of tasks and cognitive subskills, Crus I, was differentially activated in both contrasts. However, an ROI in the motor region SMC was also differentially activated in both contrasts. Furthermore, in the whole brain analysis, differential activation of cerebellar motor regions lobules VI and VIII, not far from the ROI in the lobule VIII, was observed for both internal and external monitoring. The presence of these motor regions for both internal and external monitoring might indicate sensorimotor driven, hierarchically subordinate speech motor control in addition to the hierarchically superior performance monitoring carried out in Crus I. SMC activation was previously reported for external, but not internal, monitoring (Runnqvist et al., 2021) in a lexical task. However, as the number of errors in that study did not allow distinguishing between fluent and disfluent errors, activation of SMC was likely due to articulatory errors, fitting well with the idea that this region engages in controlling speech motor implementation (e.g., Ackermann et al., 1992). Alternatively or in addition, SMC as well as the motor regions lobules VIII and VI could play a slightly different (not only hierarchically subordinate) function. For instance, the SMC is considered to be involved in the generation of proprioceptive input during internal modeling (as opposed to predictive input through Crus I; e.g., Bosco & Poppele, 2001; Tanaka et al., 2020). Yet an alternative account can be found in the directions into velocities of articulators (DIVA) model, where the superior lateral cerebellum that Crus I belongs to contributes to sensory predictions that form auditory and somatosensory targets. In turn, projections from the SMC are thought to contribute to precisely timed feedforward commands as evidenced by speech motor deficits such as ataxic dysarthria (e.g., Golfinopoulos et al., 2010). Similarly, lobule VIII has been linked to the implementation of motor sequences and to efficiency in the representation of articulatory patterns as opposed to learning a novel sequence (e.g., Orban et al., 2010; Rauschecker et al., 2008). Interestingly, learning a new motor sequence, thus taxing monitoring heavily, has been found to also engage lobule VI (e.g., Orban et al., 2010). Lobule VI was also differentially activated (though left-lateralized) for internal monitoring of a lexical variable during speech production in addition to Crus I (Runnqvist et al., 2021). A cluster identified by the whole brain analysis peaked in lobule VI, though it likely also comprised the ROI in Crus I. Collectively, these studies suggest that lobule VI might be more cognitve or transmodal than lobule VIII or SMC. Regardless, globally the current results fit the idea of two hierarchically distinct monitoring types being carried out through the cerebellum: (speech) overarching performance monitoring in Crus I (and lobule VI) and subordinate (speech) motor control in SMC (and lobule VIII).

### Sensorimotor Driven Feedback Monitoring in the Medial Frontal Cortex

We further looked into medial frontal (pre-SMA, ACC) brain regions to contrast conflict-based monitoring with sensorimotor driven monitoring of articulatory representations or sensory feedback. Here, a conflict-based account of monitoring would predict the involvement of medial frontal structures, in particular the ACC, for both internal and external monitoring. Differential pre-SMA activation was observed for both internal and external monitoring, but the ROI in the ACC was only activated for external monitoring. Regarding the pre-SMA, the whole brain analysis largely confirmed this pattern by revealing significant clusters peaking in the SMA, but presumably comprising the ROI in pre-SMA for both internal and external monitoring (see Figure 5). This pattern is both consistent with the idea of conflict-based monitoring and with the notion that the pre-SMA is involved when motor-related difficulties arise. In favor of the latter, the peak of the external monitoring contrast was slightly more anterior (closer to pre-SMA) compared to the internal monitoring contrast (Figure 5), suggesting a possible dissociation in the relative contribution of pre-SMA and SMA to internal and external monitoring. Concerning the ACC, the present findings are more consistent with a role of the targeted cingulate region in processing feedback as indicated by overt errors (as opposed to performing conflict-based monitoring). This is in line with the ACC exclusively engaging in external monitoring in Runnqvist et al. (2021), who proposed an integrative account of ACC activations for error and feedback monitoring following the proposed vocal control network put forward by Loh et al. (2020). These authors proposed that, across primates, area 44 is in charge of cognitive control of orofacial and non-speech vocal responses, and the mid-cingulate cortex is in charge of analyzing vocal non-speech feedback, driving response adaptation. Furthermore, the cognitive control of human-specific vocalization would require additional recruitment of area 45 and the pre-SMA. In this framework, it would not be the conflict that generates activation in the ACC and pre-SMA, but rather the feedback provided through the articulated error. While the present ACC pattern fits well with this explanation, the pre-SMA pattern would require assuming that the internal monitoring contrast reflects some form of sensory feedback, such as pre-articulatory proprioception. Further supporting the hypothesis of a vocal feedback control network here is activation in BA 45 (see Figure 5) just as in Runnqvist et al. (2021). Thus, the current results support the notion that the medial frontal cortex is involved in sensorimotor driven feedback monitoring. Nevertheless, a caveat to this conclusion is the considerable size and functional heterogeneity of the medial frontal cortex. Due to this, a better understanding of its contributions to monitoring will likely require both the consideration of a larger set of anatomical subregions and the use of functional localizers.

### Predictive Monitoring Supported by Temporo-Parietal Cortices

Finally, we looked into temporo-parietal (pSTG, SPT) brain regions to contrast internal modeling of speech with sensorimotor driven monitoring by auditory feedback. The ROI approach only revealed a differential pSTG activation in the internal monitoring contrast. This result differs from previous results that reported pSTG activation in conditions taxing external but not internal monitoring. However, when completing this picture with the results of the whole brain analysis, one can see that while only the internal monitoring contrast results in differential activation of BA 41, both monitoring contrasts engage BA22 (though with different laterality). A recent meta-analysis (Meekings & Scott, 2021) indicates that active regions in speech error monitoring often differ across studies, and consequently renders the functional role of the pSTG controversial. Clearly, the present results contribute to this impression of disparity, but at the same time also indicate that the temporal cortex engages in error monitoring. A systematic research approach seems necessary to increase a better understanding of the relevant variables governing the location of such monitoring related activations. The whole brain analysis further revealed a significant cluster in the inferior parietal gyrus in both contrasts, possibly comprising part of the SPT ROI. Moreover, a cluster in the superior parietal gyrus was observed in the contrast of internal monitoring. Besides possibly comprising a part of SPT, these parietal clusters are of interest because the parietal cortex has been linked to motor imagery and the retrieval of a stored internal model (e.g., Blakemore & Sirigu, 2003). A functional loop between the parietal cortex and the cerebellum, estimating the current status of the motor system throughout movement execution and allowing for predictive monitoring, has been proposed (e.g., Blakemore & Sirigu, 2003; Ito, 2008; Sokolov et al., 2017). Thus, the combined but differential activation of temporal, parietal, and cerebellar regions for internal and external monitoring suggests that their involvement goes beyond sensory feedback and speech comprehension and is more in line with proposals linking these regions to auditory and somatosensory targets and predictive internal modeling-like mechanisms in speech production (e.g., Guenther et al., 2006; Hickok, 2012, 2014; Tourville & Guenther, 2011).

### Both Internal and External Monitoring and Position Along the Cognitive-to-Motor Continuum Are Relevant in Eliciting Differential Brain Activation

Given the current results, a substantial overlap between internal and external monitoring presents that differs from prior results on the lexical level of internal monitoring (e.g., Runnqvist et al., 2021). This indicates that to some extent, the relevant variable triggering the recruitment of certain brain regions is related to the position of the representation being monitored along the cognitive-to-motor continuum (rather than to whether it takes place during speech planning or upon articulation). However, as differentiation shows, this also indicates that internal and external monitoring might play a role in the use of brain regions sustaining feedback-based mechanisms (see Sensorimotor Driven Feedback Monitoring in the Medial Frontal Cortex above). How do the current results fit the inherent assumption of existing speech production models that monitoring relies on a single overarching mechanism applied during different processing stages, before and after articulation (e.g., Levelt et al., 1999; Nozari et al., 2011; Pickering & Garrod, 2013)? The present study shows that this assumption is correct to some extent. Across all contrasts of interest, we obtained evidence for monitoring presumably carried out by predictive internal modeling (cerebellar Crus I) and possibly linked to temporal and parietal cortex (e.g., Clower et al., 2001; Glickstein, 2000; Stockert et al., 2021). However, when also considering how these findings may fit related prior literature, some of our observations could indicate a sensorimotor driven type of monitoring or control in motor regions of the cerebellum and in the medial frontal cortex. As most models of speech production agree that not all levels of speech are directly linked to sensorimotor aspects (e.g., Dell, 1988; Levelt et al., 1999; but see Fairs et al., 2021, and Strijkers, 2016, for an account of parallel and distributed processing in speech production), it is perhaps not surprising that levels directly connected to sensoimotor aspects of speech, capitalize on monitoring mechanisms based on feedback from these properties (i.e., auditory and proprioceptive feedback). An open question to be addressed in future research is whether the coexistence of an overarching and sensorimotor driven monitoring indicates complementary functions of the brain regions involved (monitoring different representations or triggered to deal with specific needs such as repairing or compensating for overt errors), or rather a hierarchical relationship ranging from implementation control to performance monitoring across nodes of a functionally coupled monitoring network.

## ACKNOWLEDGMENTS

Centre de Calcul Intensif d’Aix-Marseille is acknowledged for granting access to its high-performance computing resources. Work for this study was performed in the IRM-INT Center (UMR 7289, CNRS–Aix-Marseille University), platform member of France Life Imaging network. This work, carried out within the Institute of Convergence ILCB (ANR-16-CONV-0002), has benefited from support from the French government (France 2030), managed by the French National Agency for Research (ANR) and the Excellence Initiative of Aix-Marseille University (A*MIDEX).

## FUNDING INFORMATION

Elin Runnqvist, French National Agency for Research, Award ID: ANR-18-CE28-0013. ILCB, French National Agency for Research, Award ID: ANR-16-CONV-000X/ANR-17-EURE-00XX. France Life Imaging, French National Agency for Research, Award ID: ANR-11-INBS-0006.

## AUTHOR CONTRIBUTIONS

**Snežana Todorović**: Data curation: Lead; Formal analysis: Equal; Visualization: Lead; Writing – original draft: Supporting. **Jean-Luc Anton**: Formal analysis: Equal; Methodology: Supporting; Supervision: Supporting; Writing – original draft: Supporting. **Julien Sein**: Formal analysis: Supporting; Methodology: Supporting; Writing – original draft: Supporting. **Bruno Nazarian**: Methodology: Supporting; Software: Lead; Writing – original draft: Supporting. **Valérie Chanoine**: Data curation: Supporting; Formal analysis: Supporting; Writing – original draft: Supporting. **Birgit Rauchbauer**: Data curation: Supporting; Writing – original draft: Supporting. **Sonja A. Kotz**: Conceptualization: Supporting; Formal analysis: Supporting; Methodology: Equal; Supervision: Equal; Writing – original draft: Supporting; Writing – review & editing: Supporting. **Elin Runnqvist**: Conceptualization: Equal; Data curation: Supporting; Formal analysis: Supporting; Funding acquisition: Lead; Methodology: Equal; Project administration: Lead; Supervision: Equal; Writing – original draft: Lead; Writing – review & editing: Lead.

## DATA AVAILABILITY STATEMENT

The analysis code and data related to this article are openly available at OpenNeuro (https://doi.org/10.18112/openneuro.ds004597.v2.0.0; OpenNeuro Accession Number: ds004597; https://openneuro.org/datasets/ds004597/versions/2.0.0).

## TECHNICAL TERMS

External monitoring: Error detection processes taking place after speech becomes overt.
Internal monitoring: Error detection processes taking place during speech planning.
Phonotactically illegal sequences: Combinations of speech sounds that do not exist in the repertoire of a given language.
